# Comparison of different conditions for DNA extraction in sputum - a pilot study

**DOI:** 10.1186/s40248-018-0166-z

**Published:** 2019-01-31

**Authors:** Martina Oriano, Leonardo Terranova, Antonio Teri, Samantha Sottotetti, Luca Ruggiero, Camilla Tafuro, Paola Marchisio, Andrea Gramegna, Francesco Amati, Fabrizio Nava, Elisa Franceschi, Lisa Cariani, Francesco Blasi, Stefano Aliberti

**Affiliations:** 1 University of Milan, Department of Pathophysiology and Transplantation, Via Francesco Sforza 35, 20122 Milan, Italy; 2 Fondazione IRCCS Ca’ Granda Ospedale Maggiore Policlinico, Internal Medicine Department, Respiratory Unit and Cystic Fibrosis Adult Center, Via Francesco Sforza 35, 20122 Milan, Italy; 3 University of Pavia, Department of Molecular Medicine, Pavia, Italy; 4 Fondazione IRCCS Ca’ Granda Ospedale Maggiore Policlinico, Paediatric Highly Intensive Care Unit, Via della Commenda 9, 20122 Milan, Italy; 5 Fondazione IRCCS Ca’ Granda Ospedale Maggiore Policlinico, Cystic Fibrosis Microbiology Laboratory, Milan, Italy

**Keywords:** DNA extraction, Sputum, Microbiota, Microbiome, Sequencing

## Abstract

**Background:**

The analysis of microbiome in respiratory samples is a topic of great interest in chronic respiratory diseases. The method used to prepare sputum samples for microbiome analysis is very heterogeneous. The selection of the most suitable methodology for DNA extraction is fundamental to have the most representative data. The objective of this study was to compare different conditions for DNA extraction from sputum in adult patients with bronchiectasis.

**Methods:**

Five sputum samples from bronchiectasis patients were collected at the Policlinico Hospital in Milan, Italy. Eighteen conditions for DNA extraction were compared, including two enzyme-based (Roche and Zymo) and one beads-based (Mobio) technique. These techniques were tested with/without Dithiothreitol (DTT) and with/without lysostaphin (0.18 and 0.36 mg/mL) step. DNA was quantified, tested using Real-time PCR for 16S rDNA and *S. aureus* and, then, microbiome was evaluated.

**Results:**

Although 16S rDNA was similarly detected across all the different techniques, Roche kit gave the highest DNA yield. The lowest Ct values for Real-time PCR for *S. aureus* was identified when lysostaphin was added. Considering genera from microbiome, alpha diversity indices did not show any significant differences between techniques, while relative abundances were more similar in presence of DTT.

**Conclusions:**

None of the conditions emerged to be superior to the others even if enzyme-based kits seem to be needed in order to have a higher extraction yield.

**Electronic supplementary material:**

The online version of this article (10.1186/s40248-018-0166-z) contains supplementary material, which is available to authorized users.

## Background

Respiratory microbiome is a topic of great interest nowadays in translational research for chronic respiratory diseases [1]. Most of the published experiences on respiratory microbiome enrolled chronic obstructive pulmonary disease (COPD) and bronchiectasis patients, including those with cystic fibrosis, and sputum has been the sample most commonly used by investigators [2–5]. Sputum collection represents an easy and non-invasive strategy for studies on respiratory microbiome. So far, sequencing of microbial communities from airway samples through meta-omic approaches, mainly based on high-throughput DNA sequencing techniques, has been confined to scientific research. Although the use of respiratory microbiome analysis has not been implemented in clinical practice yet, a possible role of this technique in stratifying patients for disease severity and predicting clinical outcomes could be considered [6].

The choice of an appropriate methodology for evaluating the microbiome in sputum samples is crucial to identify the largest biodiversity as possible and to obtain reliable and comparable results. It is a precocious phase for the development of DNA extraction methods from sputum and there is an extreme heterogeneity of DNA extraction technique in literature. However, in this respect, several challenges could be identified. First, sputum is a substrate with a complex and very difficult-to-manage matrix. The use of different techniques for DNA extraction from sputum might be limited by its nature. In order to facilitate its treatment, a solubilizing agent, such as dithiothreitol (DTT), is usually used [7]. Second, epidemiological data on chronic respiratory infections in bronchiectasis and cystic fibrosis revealed the presence of hard-to-lyse bacteria, such as *Staphylococcus aureus,* leading to the possible need of an individualized strategy to enhance bacterial lysis, such as the use of enzymes able to specifically target Gram-positive bacteria [8]. Pre-treatments with lysostaphin and lysozyme, which are able to target cell wall peptidoglycan and pentaglycine bridges respectively, have been reported in literature for microbiome analysis [9, 10]. Third, sputum complex and viscous matrix might represent a limitation for broadly used DNA extraction techniques. Among them, chemical coupled with enzymatic lysis is extensively used to treat biological fluids and tissues, while mechanical disruption is usually suggested for soil or feces. Fourth, the bioinformatic approach is not standardized and several pipelines are constantly emerging that can be applied to the analysis of microbiome data generated using next-generation sequencing (NGS) techniques. Finally, largely different methodologies, including steps ranging from wet laboratory practices to in silico data analysis, have been published in literature, limiting the comparability of results across different experiences [2, 5, 11]. It is, thus, evident the importance of assessing the performance of different methods that can be applied to the analysis of microbiome in sputum samples with a special focus on the use of DTT and lysostaphin.

The objective of this pilot study was to compare different conditions, such as the use of DTT as a homogenizing agent, lytic enzymes in order to specifically target Gram-positives and DNA extraction technique in order to isolate bacterial DNA from sputum in adult patients with bronchiectasis, according to different endpoints from total DNA extraction to microbiome analysis.

## Methods

Five sputum samples were collected from five adult bronchiectasis patients followed at the Bronchiectasis Program of the Fondazione IRCCS Ca′ Granda Ospedale Maggiore Policlinico, Milan, Italy, in January 2018. Subjects signed an informed consent and gave their approval for using samples for the purpose of this study. Aliquots of at least 5 ml of sputum were collected and stored at − 80 °C for all subsequent analyses.

### DNA extraction

A total of eighteen different conditions were evaluated. Three different commercial kits for DNA extraction were considered: 1) Roche High Pure PCR Template Preparation Kit (Hoffmann – La Roche. Basilea. Switzerland); 2) Zymo Quick-DNA Universal Kit (Zymo. Irvine. CA. USA); and 3) Mobio PowerLyzer PowerSoil DNA isolation kit (Mobio. Loker Ave West. Carlsbad. CA. USA). Roche and Zymo use the combination of chemical and enzyme-based lysis, while Mobio a mechanical destruction with bead beating. Commercial kits were used according to manufacturer’s instructions. 0.1 g from each sample was extracted with the three kits in duplicate and eluted in 50ul elution buffer.

Two types of pre-treatments of the sputum samples, preceding the DNA extraction itself, were considered: the addition of dithiothreitol (DTT; Sputafluid, Biolife Italiana Srl, Italy) 10% 1:1 in volume to 0.1 g of sputum plugs and the enzymatic digestion with a combination of lysozyme at 3.6 mg/ml and lysostaphin, (at both 0.18 and 0.36 mg/ml) (Sigma-Aldrich. Saint Louis. Missouri, USA). The following six combinations of pre-treatments were performed before using each kit: a) DTT without enzymatic step; b) DTT with 3.6 mg/ml lysozyme and 0.18 mg/ml lysostaphin; c) DTT with 3.6 mg/ml lysozyme and 0.36 mg/ml lysostaphin; d) without DTT and enzymatic step; e) without DTT with 3.6 mg/ml lysozyme and 0.18 mg/ml lysostaphin; f) without DTT with 3.6 mg/ml lysozyme and 0.36 mg/ml lysostaphin. Samples were incubated at 37° for 30 min before DNA extraction [10].

DNA extraction yield for each of the eighteen conditions was measured through quantification by Quant-IT dsDNA Assay Kit. High Sensitivity and Qubit 3.0 Fluorometer (Invitrogen, Carlsbad, CA, USA). Subsequently, samples were diluted at 5 ng/μl and tested in Real Time PCR using syber green for 16S rRNA gene amplification [12]. Each sample was tested in duplicate and cycle threshold (Ct) mean and the standard deviation was considered. Real-Time PCR for *S. aureus* was conducted on DNA extracts to ensure that the addition of lysostaphin is a useful strategy to better lyse and recover the genomic DNA from *Staphylococci* [13].

### Microbiome evaluation

The V3-V4 variable regions of the 16S rRNA gene were amplified from DNA extracts using the 16S metagenomic sequencing library preparation protocol (Illumina, San Diego, CA, USA). PCR products, approximately sized 460 base pairs, were visualized using microfluidics-based gel electrophoresis on Bioanalyzer 2100 (Agilent Santa Clara, CA, USA) and then were cleaned using AMPure XP magnetic bead-based purification (Beckman Coulter, Brea. CA, USA). Sample libraries were quantified using the Qubit as reported above and then pooled in an equimolar mode. Finally, pool was sequenced on the MiSeq (Illumina, San Diego, CA, USA) sequencing platform, using a 2 × 300 cycle V3 kit and following standard Illumina sequencing protocols.

### Bioinformatic analysis

Demultiplexed paired-end reads in FASTQ format were received from the Illumina MiSeq instrument. Sequencing data were processed following the UPARSE pipeline by Robert C. Edgar [14], using USEARCH v10.0.240 [15] and VSEARCH v2.3.4 [16]. Overall run quality was checked using FastQC v0.11.2 [17] and reports were summarized using MultiQC v1.4 [18]. Quality scores dropped towards the end of the reverse reads, so they were globally trimmed at position 200 before merging with the corresponding forward reads. Parameters for paired-end reads merging were set as follows: minimum overlapping length 19 base pairs; minimum 90% identity of alignment; merged sequences length restricted to 430–480 bases. Consensus sequences from all samples were pooled together and primers were stripped from both ends. This “raw” set of merged sequences was then quality-filtered and de-replicated to obtain a subset of high-quality unique sequences to be clustered into Operational Taxonomic Units (OTUs). Sequences with more than 1 expected number of errors (EE) were discarded and singletons removed during de-replication. OTUs were clustered at 97% identity threshold. Taxonomy prediction to the genus level for OTU sequences was performed via the SINTAX algorithm [19], using the RDP training set v16 as reference database and 0.8 as confidence threshold. An OTU table was constructed by mapping the whole set of “raw” merged paired-end reads to the representative set of OTUs, using 97% identity threshold. It was then filtered - low abundance OTUs (< 0.5 overall frequency) discarded - and normalized to the same number of reads per sample. This OTU table was used for all downstream analyses. Alpha diversity was measured for each sample using different metrics (Shannon entropy and Simpson estimators). These indices were then converted to effective number of species (ENOS) [20] to be easily compared to each other.

### Study endpoints and statistical analysis

The following results have been compared across the 18 different conditions: Extracted DNA yield, Real-time PCR for 16 s rRNA gene, Diversity indices, including Shannon entropy and Simpson estimators and their conversion into ENOS. Relative abundances and Real-time PCR for *S. aureus*. Relative abundances have been compared with results of standard microbiology.

One-way ANOVA with *post hoc* Bonferroni test has been conducted on Real-Time PCR data with a significance level of 0.05. Statistical analysis was performed in the R environment.

## Results

**Fig. 1:**
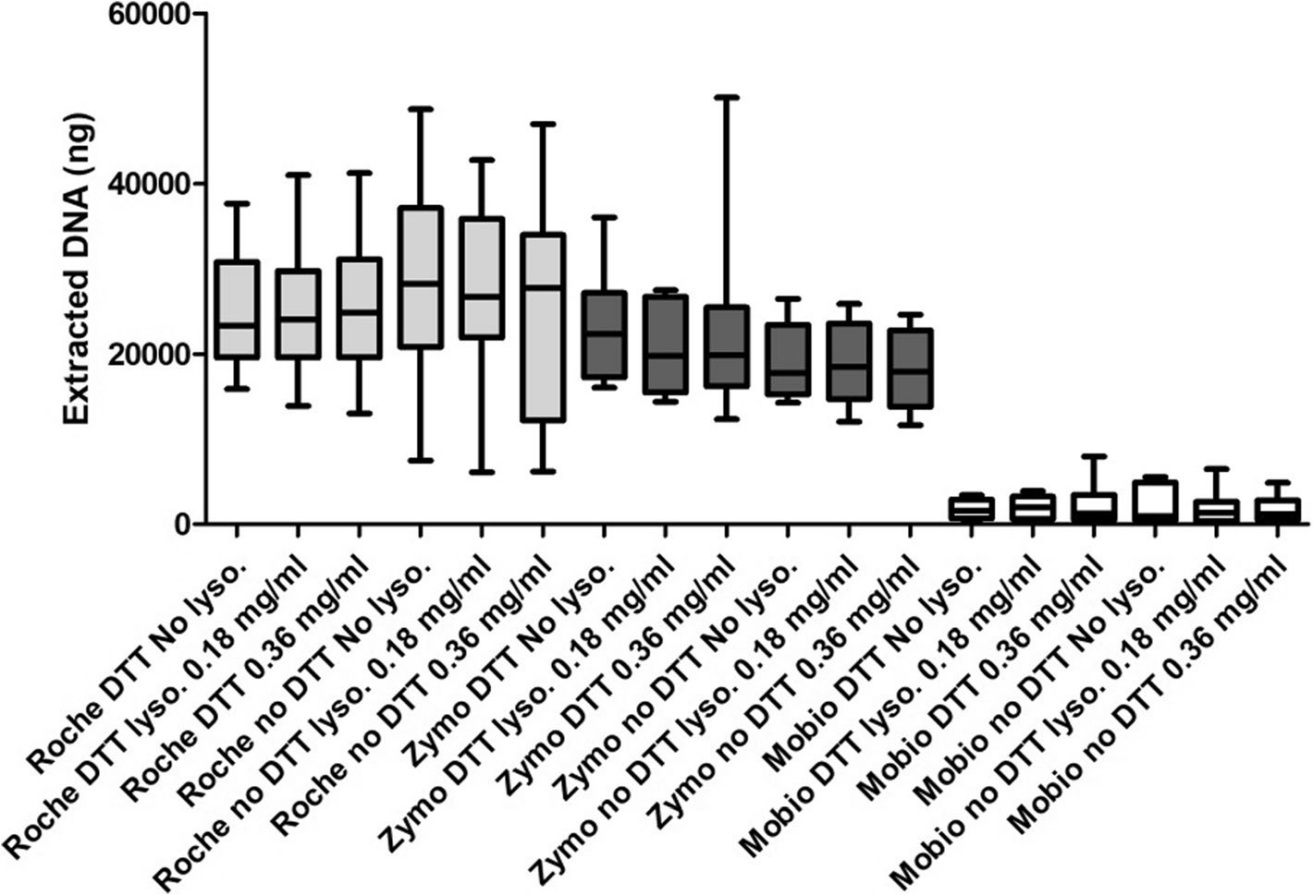
Comparison between median (IQR) levels of DNA extraction yield across 18 evaluated conditions

DNA was recovered from all the five sputum samples. A higher quantity of DNA has been extracted using Roche and Zymo kits in comparison to Mobio one; the median values of extracted DNA across the 6 different conditions for Roche, Zymo and Mobio kits were 26.062, 19.750 and 1.233 μg respectively. Detailed median (IQR) values of extracted DNA for all the 18 different conditions are reported in Fig. 1.

**Fig. 2:**
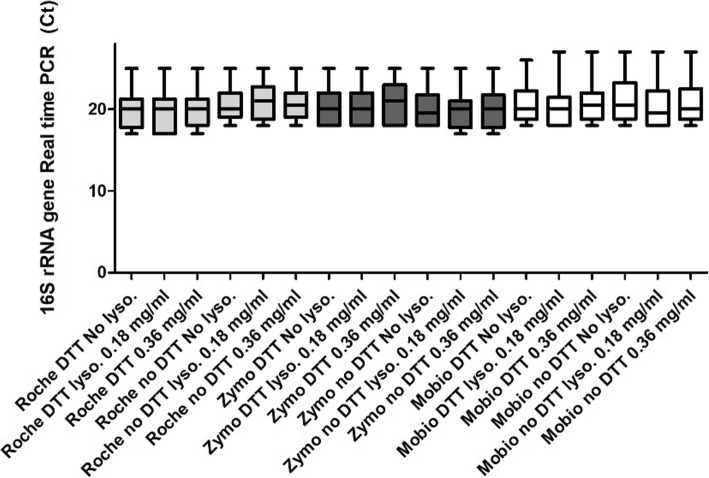
Comparison between median (IQR) levels of 16 s rRNA gene Real-Time PCR across 18 evaluated conditions

Results of the real-time PCR for 16S rRNA gene for each condition (Fig. 2), show that no significant differences across the 18 conditions (range of medians between 20 and 21 Ct).

**Fig. 3:**
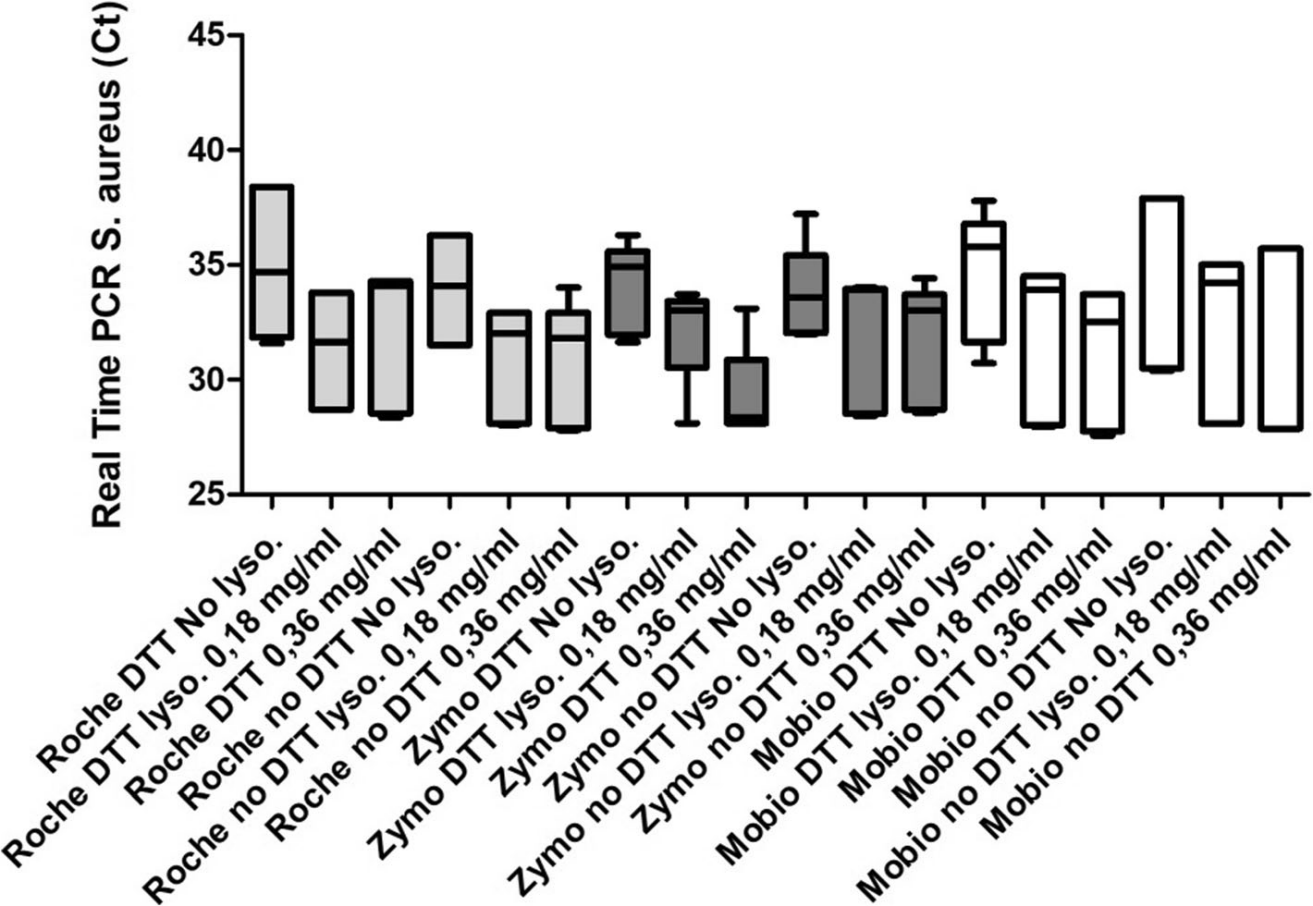
Comparison between median levels (IQR) of Real-Time PCR for *S. aureus* in sputum samples and across the 18 evaluated conditions

Real-time PCR for *S. aureus* has been performed for all samples and was positive for two of them. The addition of the enzymatic digestion step increased *Staphylococcus* DNA extraction (Fig. 3). A similar pattern can be observed through all the conditions even if no statistical evidence is present.

**Fig. 4:**
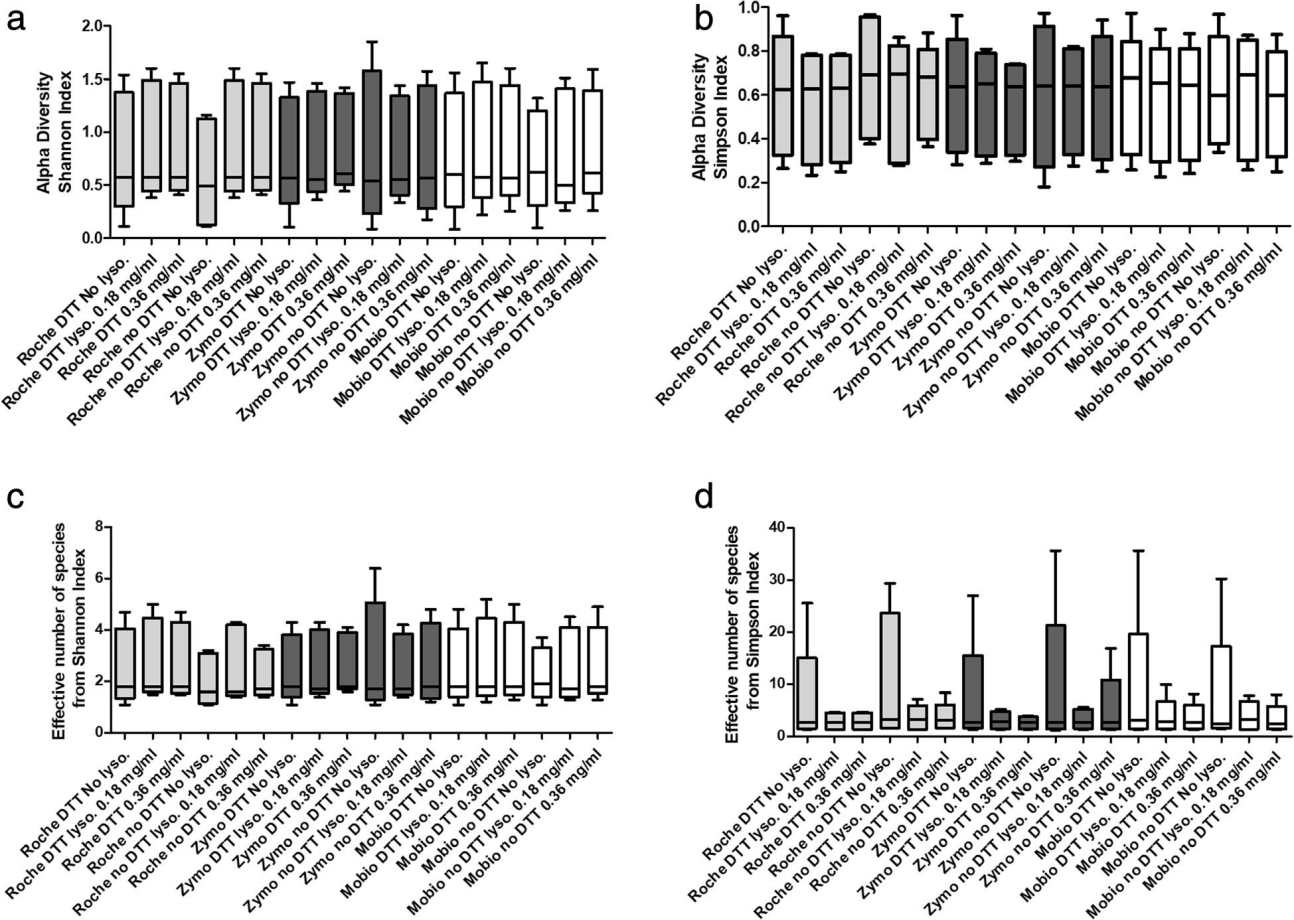
Comparison of median levels of Alpha diversity across the 18 evaluated conditions expressed as Shannon index and relative effective number of species (ENOS) (**a** and **b**), and Simpson index and relative ENOS (**c** and **d**)

In terms of alpha diversity, no significant differences have been detected among the 18 evaluated conditions, neither considering the Shannon index (range of median values: 0.492–0.574), nor the Simpson (range of median values: 0.624–0.692). Evaluation made using ENOS from Shannon index (range of median values: 1.6–1.8) and ENOS from Simpson index (range of median values: 2.7–3.3), did not show any appreciable difference (Fig. 4).

**Fig. 5:**
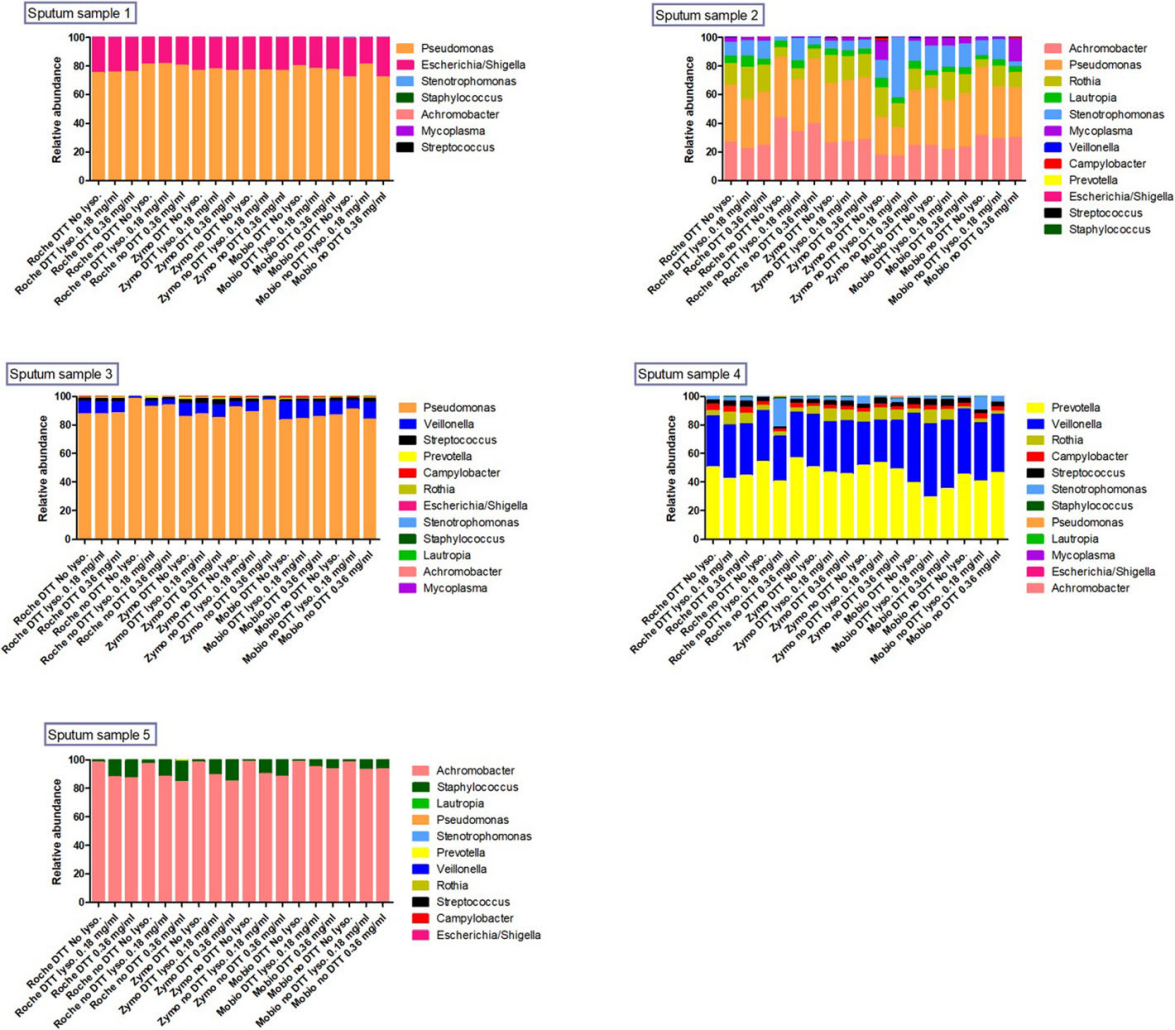
Relative abundances of bacterial genera in each of the 5 sputum samples across the 18 evaluated conditions

Relative abundances at the genus level of identified bacteria in each sputum sample across the 18 evaluated conditions are reported in Fig. 5**.** Microbiome composition is very different across different samples. Sputum samples presenting few genera have very similar data, while in the presence of a higher number of bacterial genera (Patient 2 and 4) differences emerge when comparing the same condition with and without DTT. In sputum sample 5, where only two genera are present, there is an increase of *Staphylococci* presence in samples treated with lysostaphin and lysozyme. Relative abundances of the four most abundant bacteria detected in each sputum sample across the 18 evaluated conditions are reported in (Additional file 1: Table A).

## Discussion

The major finding of the present experience is that the 18 different conditions evaluated seem to equally perform in extracting DNA from sputum samples in terms of Real-Time PCR for 16 s rRNA gene, alpha diversity and relative abundances of bacterial genera. However, the DNA extraction yield seems to be higher if Roche and Zymo kits are used in comparison to the Mobio one.

The use of enzymatic techniques seems to allow a higher DNA extraction yield than mechanical ones. A high yield might be preferred for 16 s rRNA sequencing because sputum is a matrix with a high percentage of human DNA. For this reason, in order to prepare libraries with an acceptable bacterial DNA amount and more representative for the microbiota of the sample, the starting amount of DNA loaded into PCR need to be high. In order to overcome this limitation, it is possible to either increase the amount of sputum collected by patients or, in case of a limited amount of sputum sample, select a technique able to obtain a high yield of DNA (such as Roche or Zymo kits).

Both the evaluated pre-treatments seem to play a crucial role. The addition of lytic enzymes is important to enhance *S. aureus* DNA extraction; however, an increase of lysostaphin concentration over 0.18 mg/mL seems not to increase the performance of the kit to extract DNA. These results, first identified through Real-Time PCR for *S. aureus*, are confirmed by an increase in *Staphylococcus* genus identification through sequencing. The addition of DTT has a role in improving data reproducibility. Through sequencing, we saw that the presence of DTT seemed to be able to better homogenize sputum samples, leading to more reproducible results in terms of bacterial genera detected, and at the same time seems not to affect the efficiency of DNA extraction. Relative abundances in *sputa* presenting a large number of bacterial genera are very similar in conditions with DTT, while they vary greatly but very different when it is absent. In sputum sample 2 there are differences in extraction of *Stenotrophomonas* genus, greatly improved in absence of DTT, presence of 0.18 mg/ml lysostaphin and treated with Zymo kit. Comparing this result with the same condition, in presence of DTT we might speculate that the difference is given by sampling. DTT enable the release of bacteria entrapped into sputum matrix. For this reason, the repetition of the analysis on the same sample leads to comparable results. If a homogenization step is not present, differences given by sampling can be present.

This study has some limitations. First, different interventions, such as the use of DTT and lysostaphin, have been performed at the same time, while no sequential approach has been used. Although this might help us in evaluating possible synergies between variables, difficulties in data interpretation might be present without a special focus on a single variable. Second, the monocentric design, the low number of samples collected and the fact that no patients other than bronchiectasis have been enrolled might interfere with the generalizability of our results. Third, only three kits have been considered, although others are on the market. Finally, our study did not take into account the possible presence of initial contamination of commercial kits.

This is the first pilot study that tried to address relevant methodological questions in microbiome analysis of sputum samples and took into consideration not only a comparison among different commercial kits but also different pre-treatments of sputum samples. Furthermore, the performance of 18 different conditions for DNA extraction from sputum has been evaluated considering different endpoints. Real-time PCR for both 16 s rRNA gene allowed us to understand if all methods were able to extract bacterial DNA with the same efficiency; Real-time PCR for *S. aureus* has been performed in order to evaluate lytic enzymes activity; Microbiome analysis considering alpha diversity indices and relative abundances.

## Conclusions

The use of a *unique* method for DNA extraction and microbiome analysis of sputum samples is very important in translational research and could represent a step forward in the introduction of microbiome analysis in clinical practice. A homogeneity of methods for microbiome analysis is needed as well as data coming from different centers which are needed to improve the reproducibility of the method. These data should be important for the development of new methods in order to have an improvement of DNA extraction techniques for microbiome analysis.

None of the 18 evaluated conditions seems to be superior to the other ones in extracting DNA from sputum samples, although a higher amount of extracted DNA could be obtained using enzyme-based commercial kits. Pre-treatments with lysostaphin, lysozyme and DTT seem to be necessary in order to have the most representative microbiome evaluation possible. Further studies will be necessary in order to confirm our data. Moreover, these preliminary data show that neither synergic nor interfering effect is present between variables. Punctual evaluation of variables in an independent way should be needed in order to better address this issue. The hypothesis of evaluating microbiome from the same sputum samples in accordance with the same standard operating procedures across different international centers will be considered in case of multicenter studies on microbiome analysis will be designed in the next future.

## Additional file

Additional file 1: **Table A.** Relative abundances of the four most abundant bacteria detected in each sputum sample across the 18 evaluated conditions (in percentage). (DOCX 27 kb)

## Abbreviations

COPD: Chronic obstructive pulmonary disease
Ct: Cycle threshold
DTT: Dithiothreitol
EE: Expected number of errors
ENOS: Effective number of species
NGS: Next-generation sequencing
OTUs: Operational Taxonomic Units
