# Supplementary material for: Comparison of different conditions for DNA extraction in sputum - a pilot study
Source: Multidiscip Respir Med. 2019 Jan 31;14:6. doi: 10.1186/s40248-018-0166-z (PMC6357456; doi:10.1186/s40248-018-0166-z)
Supplement: Supplementary file 1 — Table A. Relative abundances of the four most abundant bacteria detected in each sputum sample across the 18 evaluated conditions (in percentage). (DOCX 27 kb) [file 40248_2018_166_MOESM1_ESM.docx]

**ADDITIONAL FILE**

**Table A.** Relative abundances of the four most abundant bacteria detected in each sputum sample across the 18 evaluated conditions (in percentage). The four most abundant genera have been reported while the remaining were included as others

| **Patient 1** |  |  |  |  |  |
| --- | --- | --- | --- | --- | --- |
| Genus | **Pseudomonas** | **Escherichia/**  **Shigella** | **Stenotrophomonas** | **Staphylococcus** | **Others** |
| Roche DTT No lyso. | 75 | 24.8 | 0.18 | 0 | 0 |
| Roche DTT lyso. 0.18 mg/ml | 75.6 | 24.1 | 0.14 | 0.16 | 0 |
| Roche DTT 0.36 mg/ml | 75.9 | 23.8 | 0.22 | 0.14 | 0 |
| Roche no DTT No lyso. | 81 | 18.9 | 0.08 | 0 | 0 |
| Roche no DTT lyso. 0.18 mg/ml | 81.3 | 18.6 | 0.04 | 0.02 | 0 |
| Roche no DTT 0.36 mg/ml | 80.2 | 19.6 | 0.1 | 0.12 | 0 |
| Zymo DTT No lyso. | 76.6 | 23.2 | 0.2 | 0 | 0 |
| Zymo DTT lyso. 0.18 mg/ml | 77.7 | 21.9 | 0.24 | 0.1 | 0.04 |
| Zymo DTT 0.36 mg/ml | 76.6 | 23 | 0.2 | 0.18 | 0 |
| Zymo no DTT No lyso. | 76.9 | 23.1 | 0 | 0 | 0 |
| Zymo no DTT lyso. 0.18 mg/ml | 76.9 | 23 | 0.04 | 0.08 | 0 |
| Zymo no DTT 0.36 mg/ml | 76.6 | 23.1 | 0.22 | 0.14 | 0 |
| Mobio DTT No lyso. | 80 | 19.9 | 0.14 | 0.02 | 0 |
| Mobio DTT lyso. 0.18 mg/ml | 77.9 | 21.9 | 0.16 | 0.06 | 0 |
| Mobio DTT 0.36 mg/ml | 77.3 | 22.4 | 0.14 | 0.16 | 0.02 |
| Mobio no DTT No lyso. | 72.2 | 27.2 | 0.52 | 0.02 | 0 |
| Mobio no DTT lyso. 0.18 mg/ml | 81.1 | 18.6 | 0.16 | 0.12 | 0 |
| Mobio no DTT 0.36 mg/ml | 72.2 | 27.4 | 0.24 | 0.16 | 0 |
|  |  |  |  |  |  |
| **Patient 2** |  |  |  |  |  |
| Genus | Achromobacter | Pseudomonas | Rothia | Stenotrophomonas | Others |
| Roche DTT No lyso. | 26.4 | 39.9 | 15 | 9.56 | 9.1 |
| Roche DTT lyso. 0.18 mg/ml | 22 | 34 | 22.8 | 10.6 | 10.6 |
| Roche DTT 0.36 mg/ml | 24.4 | 36.8 | 19 | 12.5 | 7.34 |
| Roche no DTT No lyso. | 43.5 | 42.3 | 6.68 | 2.8 | 4.68 |
| Roche no DTT lyso. 0.18 mg/ml | 34 | 35.8 | 7.88 | 15.4 | 6.94 |
| Roche no DTT 0.36 mg/ml | 39.4 | 44.9 | 7.18 | 5.02 | 3.5 |
| Zymo DTT No lyso. | 26.2 | 41.1 | 19.8 | 5.84 | 7 |
| Zymo DTT lyso. 0.18 mg/ml | 26.9 | 42.5 | 16.9 | 7.06 | 6.62 |
| Zymo DTT 0.36 mg/ml | 28.3 | 43 | 16.6 | 6.22 | 5.84 |
| Zymo no DTT No lyso. | 17.4 | 26.5 | 20.5 | 12.3 | 23.4 |
| Zymo no DTT lyso. 0.18 mg/ml | 16.8 | 20.1 | 16.2 | 42.2 | 4.64 |
| Zymo no DTT 0.36 mg/ml | 24.1 | 38.3 | 14.7 | 14.1 | 8.74 |
| Mobio DTT No lyso. | 24.4 | 39.5 | 8.8 | 17.4 | 9.88 |
| Mobio DTT lyso. 0.18 mg/ml | 21.4 | 34.1 | 19.6 | 14.6 | 10.28 |
| Mobio DTT 0.36 mg/ml | 23 | 37.3 | 13.3 | 16.5 | 9.92 |
| Mobio no DTT No lyso. | 31.4 | 47.5 | 5.26 | 10.4 | 5.46 |
| Mobio no DTT lyso. 0.18 mg/ml | 28.9 | 36.3 | 14.4 | 14 | 6.36 |
| Mobio no DTT 0.36 mg/ml | 29.6 | 34.9 | 10.5 | 3.28 | 21.72 |
|  |  |  |  |  |  |
| Patient 3 |  |  |  |  |  |
| Genus | Pseudomonas | Veillonella | Streptococcus | Prevotella | Others |
| Roche DTT No lyso. | 87.5 | 8.9 | 2.14 | 0.7 | 0.74 |
| Roche DTT lyso. 0.18 mg/ml | 87.5 | 8.5 | 2.28 | 0.84 | 0.9 |
| Roche DTT 0.36 mg/ml | 88.4 | 7.56 | 2.32 | 0.76 | 1 |
| Roche no DTT No lyso. | 98.3 | 1.04 | 0.38 | 0.14 | 0.16 |
| Roche no DTT lyso. 0.18 mg/ml | 92.8 | 3.66 | 1.74 | 0.68 | 1.16 |
| Roche no DTT 0.36 mg/ml | 93.8 | 3.66 | 1.42 | 0.66 | 0.46 |
| Zymo DTT No lyso. | 85.8 | 8.96 | 2.98 | 1.3 | 0.98 |
| Zymo DTT lyso. 0.18 mg/ml | 87.4 | 7.64 | 3.26 | 0.76 | 0.9 |
| Zymo DTT 0.36 mg/ml | 84.9 | 8.86 | 3.78 | 1.14 | 1.34 |
| Zymo no DTT No lyso. | 92.3 | 3.78 | 2.16 | 0.4 | 1.4 |
| Zymo no DTT lyso. 0.18 mg/ml | 89 | 6.62 | 2.2 | 0.68 | 1.48 |
| Zymo no DTT 0.36 mg/ml | 97 | 1.5 | 1.12 | 0.08 | 0.32 |
| Mobio DTT No lyso. | 83.4 | 12.6 | 1.58 | 0.6 | 1.82 |
| Mobio DTT lyso. 0.18 mg/ml | 84.1 | 12.2 | 1.68 | 0.36 | 1.64 |
| Mobio DTT 0.36 mg/ml | 85.5 | 10 | 2.42 | 0.84 | 1.24 |
| Mobio no DTT No lyso. | 86.8 | 9.7 | 2.08 | 0.42 | 0.98 |
| Mobio no DTT lyso. 0.18 mg/ml | 90.8 | 6.18 | 1.52 | 0.22 | 1.32 |
| Mobio no DTT 0.36 mg/ml | 83.8 | 12 | 2.32 | 0.5 | 1.36 |
|  |  |  |  |  |  |
| Patient 4 |  |  |  |  |  |
| Genus | Prevotella | Veillonella | Rothia | Campylobacter | Others |
| Roche DTT No lyso. | 50.2 | 35.9 | 3.76 | 4.2 | 5.94 |
| Roche DTT lyso. 0.18 mg/ml | 42.3 | 37.4 | 8.92 | 3.92 | 7.42 |
| Roche DTT 0.36 mg/ml | 44.3 | 36.2 | 7.3 | 4.18 | 8.04 |
| Roche no DTT No lyso. | 54 | 35.8 | 3.58 | 2.86 | 3.78 |
| Roche no DTT lyso. 0.18 mg/ml | 40.3 | 31.5 | 3.02 | 1.82 | 23.32 |
| Roche no DTT 0.36 mg/ml | 56.8 | 32 | 2.98 | 3.16 | 5.06 |
| Zymo DTT No lyso. | 50.4 | 36.8 | 5.34 | 2.1 | 5.36 |
| Zymo DTT lyso. 0.18 mg/ml | 46.6 | 35.5 | 8.78 | 2.58 | 6.58 |
| Zymo DTT 0.36 mg/ml | 45.5 | 37.2 | 7.4 | 2.64 | 7.24 |
| Zymo no DTT No lyso. | 51.4 | 30.2 | 7.12 | 2.44 | 8.86 |
| Zymo no DTT lyso. 0.18 mg/ml | 53.4 | 29.7 | 8.7 | 2.38 | 5.82 |
| Zymo no DTT 0.36 mg/ml | 49 | 34 | 7.14 | 2.12 | 7.7 |
| Mobio DTT No lyso. | 39.3 | 48.7 | 2.88 | 2.8 | 6.36 |
| Mobio DTT lyso. 0.18 mg/ml | 29.3 | 51.3 | 9.48 | 2.88 | 7 |
| Mobio DTT 0.36 mg/ml | 35.2 | 47.9 | 7.46 | 2.12 | 7.3 |
| Mobio no DTT No lyso. | 45.1 | 45.9 | 1.26 | 2.8 | 4.92 |
| Mobio no DTT lyso. 0.18 mg/ml | 40.4 | 41 | 2.46 | 3.6 | 12.48 |
| Mobio no DTT 0.36 mg/ml | 46.4 | 40.9 | 2.24 | 2.92 | 7.56 |
|  |  |  |  |  |  |
| Patient 5 |  |  |  |  |  |
| Genus | Achromobacter | Staphylococcus | Lautropia | Pseudomonas | Stenotrophomonas |
| Roche DTT No lyso. | 98 | 1.64 | 0.34 | 0 | 0.02 |
| Roche DTT lyso. 0.18 mg/ml | 87.9 | 11.9 | 0.22 | 0 | 0 |
| Roche DTT 0.36 mg/ml | 87.2 | 12.4 | 0.28 | 0.14 | 0.06 |
| Roche no DTT No lyso. | 97.1 | 2.34 | 0.54 | 0 | 0 |
| Roche no DTT lyso. 0.18 mg/ml | 88 | 11.6 | 0.4 | 0.02 | 0 |
| Roche no DTT 0.36 mg/ml | 84.5 | 14.2 | 0.34 | 0 | 0.92 |
| Zymo DTT No lyso. | 98.1 | 1.5 | 0.36 | 0 | 0.02 |
| Zymo DTT lyso. 0.18 mg/ml | 89.2 | 10.4 | 0.32 | 0.02 | 0.02 |
| Zymo DTT 0.36 mg/ml | 84.9 | 14.8 | 0.32 | 0 | 0 |
| Zymo no DTT No lyso. | 98.6 | 1.14 | 0.28 | 0 | 0.02 |
| Zymo no DTT lyso. 0.18 mg/ml | 90.1 | 9.68 | 0.22 | 0 | 0 |
| Zymo no DTT 0.36 mg/ml | 88.1 | 11.4 | 0.5 | 0 | 0 |
| Mobio DTT No lyso. | 98.6 | 1.32 | 0.12 | 0 | 0 |
| Mobio DTT lyso. 0.18 mg/ml | 94.7 | 5.08 | 0.24 | 0 | 0 |
| Mobio DTT 0.36 mg/ml | 93.5 | 6.36 | 0.16 | 0 | 0 |
| Mobio no DTT No lyso. | 98.3 | 1.46 | 0.22 | 0.02 | 0 |
| Mobio no DTT lyso. 0.18 mg/ml | 93.1 | 6.7 | 0.16 | 0 | 0 |
| Mobio no DTT 0.36 mg/ml | 93.3 | 6.46 | 0.24 | 0 | 0 |
